# Contamination-controlled high-throughput whole genome sequencing for influenza A viruses using the MiSeq sequencer

**DOI:** 10.1038/srep33318

**Published:** 2016-09-14

**Authors:** Hong Kai Lee, Chun Kiat Lee, Julian Wei-Tze Tang, Tze Ping Loh, Evelyn Siew-Chuan Koay

**Affiliations:** 1 Department of Laboratory Medicine, National University Hospital, National University Health System, Singapore; 2 Department of Infection, Immunity, Inflammation, University of Leicester, Leicester, UK; 3 Clinical Microbiology, Leicester Royal Infirmary, Leicester, UK; 4 Department of Pathology, Yong Loo Lin School of Medicine, National University of Singapore, Singapore

## Abstract

Accurate full-length genomic sequences are important for viral phylogenetic studies. We developed a targeted high-throughput whole genome sequencing (HT-WGS) method for influenza A viruses, which utilized an enzymatic cleavage-based approach, the Nextera XT DNA library preparation kit, for library preparation. The entire library preparation workflow was adapted for the Sentosa SX101, a liquid handling platform, to automate this labor-intensive step. As the enzymatic cleavage-based approach generates low coverage reads at both ends of the cleaved products, we corrected this loss of sequencing coverage at the termini by introducing modified primers during the targeted amplification step to generate full-length influenza A sequences with even coverage across the whole genome. Another challenge of targeted HTS is the risk of specimen-to-specimen cross-contamination during the library preparation step that results in the calling of false-positive minority variants. We included an in-run, negative system control to capture contamination reads that may be generated during the liquid handling procedures. The upper limits of 99.99% prediction intervals of the contamination rate were adopted as cut-off values of contamination reads. Here, 148 influenza A/H3N2 samples were sequenced using the HTS protocol and were compared against a Sanger-based sequencing method. Our data showed that the rate of specimen-to-specimen cross-contamination was highly significant in HTS.

High-throughput sequencing (HTS) technologies allow rapid and relatively inexpensive acquisition of high-resolution genomic data, and are increasingly used for full genome analysis in microbiological studies. The advent of HTS makes it possible to sequence the rapidly evolving influenza A viral genomes more frequently, to supplement the epidemiological surveillance programs for the six monthly vaccine formulation and drug resistance monitoring^1^.

An amplicon-based HTS library preparation kit, Nextera XT DNA library preparation (Illumina Inc., CA, USA), employs an enzymatic process that cleaves DNA amplicons into fragments of approximately 300 base-pairs while integrating transposon sequences onto both 5′- and 3′-ends of the fragments. Subsequent sequencing of the tagmented fragments produces reduced coverage at the 3′- and 5′-termini as the transposases are less favorable to bind to the very ends of the amplicons (Fig. 1a)^2^. We resolved this issue by optimizing the DNA library generation by introducing modified primers to produce full-length influenza A sequences with even coverage, from one end to the other.

Most of the current HTS applications for human gene mutations focus on detecting specific variants such as point mutations, gene deletions or duplications^3^,^4^, without the need to perform whole-genome sequencing of the sample, because of the accuracy and proof-reading efficiency inherent in the human DNA replication process. In contrast, for infectious disease monitoring, obtaining an accurate full-length sequence of the causal infective organism is important for phylogenetic or surveillance studies, and taxonomy assignment, especially for fast-evolving RNA viruses. In such genetically labile viruses, the validity of variant calling by HTS is critical for all nucleotide positions across the whole genome, rather than focusing only on certain mutational hotspots.

Evidence of erroneous variant calling due to nucleic acid contamination have been found in several clinical and research applications utilizing HTS^5^,^6^,^7^,^8^,^9^. Most amplicon-based (targeted) HTS methods use two sequential polymerase chain reactions (PCRs) to rapidly generate sequencing-ready libraries for high read coverage of the targets^3^,^4^. Pipetting and transferring the amplified products of the first targeted PCR, when setting up the second indexing/barcoding PCR, carry a high risk of between-sample contamination^10^.

Here, a method to minimize erroneous variant calling from contamination during an amplicon-based HTS library preparation (i.e. Nextera XT DNA Library Preparation kit) for full influenza genome sequencing, was developed and compared against a Sanger-based sequencing method. To test the validity of the variant calls and viral genomes obtained from this contamination-controlled HTS method, we compared a total of 1099 complete gene sequences from 148 influenza A/H3N2 viruses derived from HTS on the MiSeq sequencer (Illumina) with those from a Sanger-based sequencer, ABI 3130xl genetic analyzer (Applied Biosystems, CA, USA). The 1099 sequences were comprised of 8 gene segments of the influenza A/H3N2 viruses, with different lengths and variable mutation rates for all gene segments.

## Results

### Genome-wide amplification for even genome coverage

**Figure 1:**
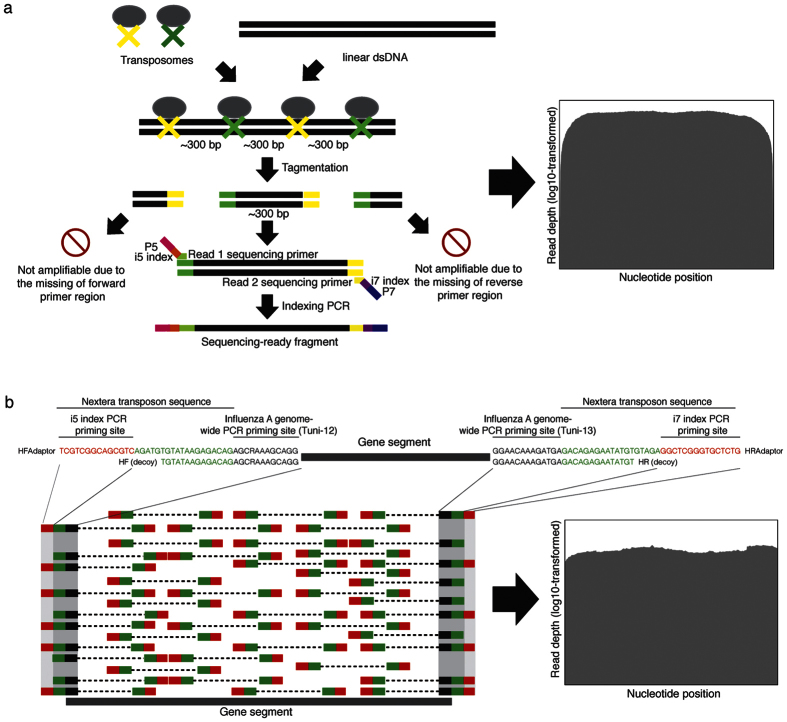
Optimization of the DNA library generation by introducing decoy PCR primers to produce full-length influenza A/H3N2 sequences with even coverage. (**a**) The existing protocol for Nextera XT library preparation on targeted amplicons. Low sequencing read depths were typically found at both 5′ and 3′ ends of the amplicons. (**b**) The direct integration of the transposon sequences to the Tuni-12 and Tuni-13 primer sequences and inclusion of decoy primers during the initial genome-wide PCR before the Nextera XT library preparation. This increases the read depths at both ends of the amplicons.

Influenza A virus genomic segmental RNAs have 12 nucleotides at the 3′ terminus and 13 nucleotides at the 5′ terminus that are conserved and complementary, namely Uni-12 and Uni-13, respectively^11^. The inclusion of Tuni-12 (i.e. Sequence complementary to Uni-12) and Tuni-13 (i.e. Uni-13) sequences allows genome-wide amplification and sequencing for most of the influenza A subtypes^11^, including: A/H1N1/2009, A/H3N2, seasonal A/H1N1, as well as avian influenza A/H7N9 and A/H5N1 subtypes. To achieve even coverage across the entire genome sequence, complete Nextera transposon sequences (Illumina) were added as overhanging regions adjacent to the 5′-end of Tuni-12 and Tuni-13 sequences to form the first set of PCR primers, i.e. HFAdaptor and HRAdaptor (Fig. 1b). Additionally, to increase the assay sensitivity, partial Nextera transposon sequences were added to the 5′-end of Tuni-12 and Tuni-13 sequences to form decoy PCR primers, i.e. HF and HR (Fig. 1b). The lack of i5 and i7 index PCR priming sites in the partial Nextera transposon sequences prohibit index PCR amplification of the 3′- and 5′ termini of amplicons generated by the decoy PCR primers. All the primer sequences used in this study are provided in Fig. 1b. A primer titration experiment, performed during the initial experimental set-up, produced an even coverage of reads in each of the gene segments. The shorter 5′-overhang regions also helped to increase the PCR sensitivity in amplifying the genome sequences, from 10^8^ RNA copies/PCR to 10^4^ RNA copies/PCR (Supplementary Fig. 1).

### Selection of the best-matching reference sequence

Accuracy and efficiency of HTS read alignments are dependent on the degree of matching of the target sequence to the reference sequence. Selecting the most appropriate reference sequence for read mapping is crucial in virology work, especially for fast-evolving RNA viruses, as the genomic differences can be significant even within similar strain populations^12^,^13^. This study first performed a *de novo* contig assembly of the sequencing reads for a sample, followed by mapping of the contigs to a local database of all influenza A virus sequences downloaded from the NCBI Influenza Virus Resource (Last accessed: 25 November 2015). This approach ensured the best-matching reference sequences for subsequent read mapping and variant calling. Also, it provided immediate strain/subtype identification for the virus causing an outbreak. The best-matching reference strain identity can be obtained from the FASTA defline assigned to the FASTA sequence of the best-matching reference.

Another novel feature of our experimental design was the replacement of all degenerate sequences of the best-matching reference gene segments derived above with non-degenerate sequences (i.e. A, T, C, or G), using an in-house developed algorithm. Such replacement ensured the reference nucleotide in the.vcf file generated by the GATK variant caller software^14^,^15^,^16^ will always be non-degenerate nucleotides. The in-house developed algorithm for generating final consensus sequence for a sample required non-degenerate reference nucleotides from the.vcf file.

### Artificial nucleotide replacement of the best-matching reference sequence

The read depths of the entire sequence region were critical for contamination filtering developed in this study. The GATK variant caller used in this study will not produce a variant call when there is no mismatch found in a region against the reference sequence, regardless of the read depth of the sequence. To ensure regional filtering of contamination across the entire gene segment, a nucleotide replacement was performed every 150^th^ nucleotide starting from the first nucleotide of the best-matching reference sequence, using the in-house developed algorithm. As such, the read depth of the entire gene length will be estimated in regional manner, according to read depths called for the variants introduced artificially. The final consensus sequences for the amplicons were generated based on the best-matching reference sequence, according to the noise-filtered variant calls mentioned below.

### Analysis of contamination reads

**Figure 2:**
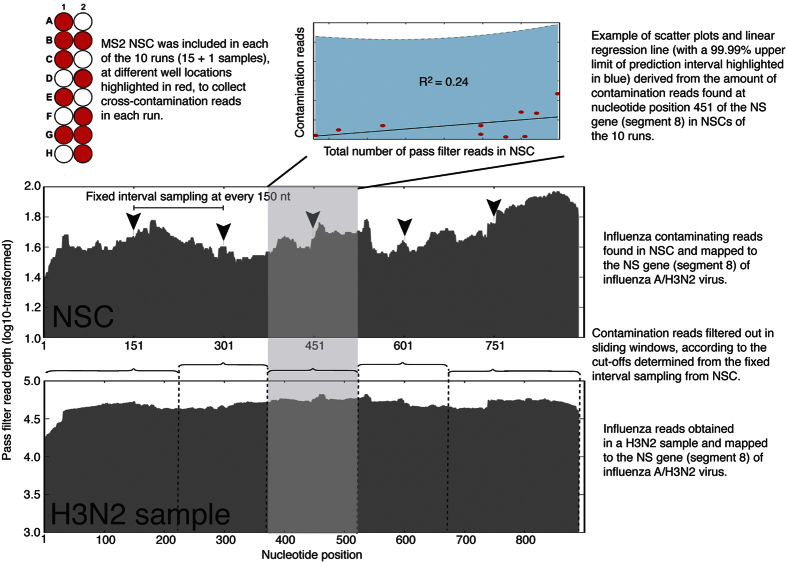
**C**ontamination noise analysis using in-run Negative System Controls (NSCs). Only non-structural (NS) gene (segment 8) of the influenza A/H3N2 virus is used for illustration purpose. The depth of coverage for the contaminating NS gene found in the NSCs included in the 10 separate runs were recorded at every 150^th^ nucleotide position starting from the first nucleotide of the NS gene onwards. As shown here, the number of contamination reads at the 451^th^ nucleotide position was plotted against the number of total pass-filter reads in the NSC. The linear regression model at this nucleotide position of NS gene had the lowest R^2^ value (0.24) among all from all 8 genes (ranging from 0.24 to 0.70). The upper limits of 99.99% prediction intervals derived from the regression model of current 451^th^ nucleotide position were adopted as cut-off value of contamination reads for nucleotide sequence located in the grey-highlighted region. All base calls with read depths below the cut-off values were considered as contamination reads and excluded from further analysis.

The HTS protocol was applied initially on 15 influenza A/H3N2 samples, using the Nextera XT DNA Library Prep Kit. Amplicons of the RNA replicase gene of the MS2 bacteriophage (1130 bp) were included as a Negative System Control (NSC) in a separate reaction well in each run, to collect cross-contaminating reads. The full influenza A/H3N2 genomes were successfully obtained for these 15 samples. The NSC should contain only amplicons of the RNA replicase gene of the MS2 bacteriophage. In the event of any cross-contamination by influenza A/H3N2 amplicons from any surrounding well, they would be identifiable by mapping all the NSC reads against the influenza A/H3N2 reference sequence (Fig. 2).

Subsequently, the genome sequencing of 148 influenza A/H3N2 clinical respiratory samples were subjected to the above protocol using a semi-automated library preparation protocol optimized on the Sentosa SX101 liquid handling machine (Vela Diagnostics, Singapore). All the 148 samples were sequenced in a total of 10 runs. The NSCs were placed in different well positions of the 10 library preparation plates (Fig. 2). The viral loads of the 148 samples ranged from 3.5 × 10^2^ to 6.6 × 10^7^ viral copies/μL of RNA extract (median = 1.7 × 10^5^ viral copies/μL), quantitated using a clinically validated real-time influenza A/B screening assay^17^ (Supplementary Dataset 1).

The highest amount of contaminating reads found for gene segments 1 to 8 in the NSCs included in the 10 separate runs were 159, 76, 188, 88, 139, 74, 706, and 178, respectively. The depth of coverage for the contaminating influenza genome found in the 10 NSCs were surveyed at a fixed interval of 150 nucleotides, starting from the first nucleotide of the reference sequence onwards, for all the 8 different gene segments (Fig. 2). The number of contamination reads (representing the depth of coverage) were plotted against the number of total pass-filter reads in the NSC. The upper limits of 99.99% prediction intervals derived from each regression model of the surveyed depth of contaminating coverage were adopted as cut-off values of contamination reads (Fig. 2). This had the effect of assigning the appropriate cut-off value that is dependent on the number of total pass-filter reads, instead of a universal cut-off. This optimizes the filtering process, where a high total pass-filter reads will be assigned a higher cut-off value, and vice-versa. Of note, a 99.99% prediction interval is an estimate of an interval in which future observations will fall, with a probability of 99.99%, given what has already been observed^18^, i.e. previous sequencing runs in this case.

All base calls with read depths below the cut-off values were considered as contamination reads and excluded from further analysis. The median R^2^ values derived from least squares regressions for every 150^th^ nucleotide position for gene segments 1 to 8 were 0.48, (range: 0.36–0.67), 0.49 (range: 0.31–0.56), 0.38 (range: 0.27–0.57), 0.50 (range: 0.30–0.67), 0.55 (range: 0.44–0.65), 0.50 (range: 0.38–0.70), 0.57 (range: 0.53–0.63), 0.30 (range: 0.24–0.31), respectively (Supplementary Fig. S2). Seventy percent of p-values associated with the R^2^ were statistically significant, i.e. lower than 0.05 (Supplementary Dataset 2).

### Full-length gene sequences

After excluding the contamination reads, 130 (88%) polymerase basic 2 (PB2, segment 1), 130 (88%) polymerase basic 1 (PB1, segment 2), 136 (92%) polymerase acidic (PA, segment 3), 138 (93%) hemagglutinin (HA, segment 4), 142 (96%) nucleoprotein (NP, segment 5), 140 (95%) neuraminidase (NA, segment 6), 142 (96%) matrix protein (MP, segment 7), and 143 (97%) nonstructural (NS, segment 8) genes from the 148 samples retained their full sequences (Table 1). All the sequences were compared against those derived from a Sanger sequencing method^19^, except for segment 1 of the influenza A/Singapore/H2013.901/2013(H3N2) strain and segment 3 of the influenza A/Singapore/H2012.779/2012(H3N2) strain, where the Sanger sequencing failed. The GenBank accession numbers of the genomes and their respective sample viral loads are provided in Supplementary Dataset 1. Notably, for HTS using the MiSeq instrument, gene segments with sequence lengths >2000 nucleotides (segments 1 to 3) produced significantly fewer numbers of fully concordant sequences compared to gene segments with sequence lengths <2000 nucleotides (segments 4 to 8; p value < 0.0001) (Table 1).

**Figure 3:**
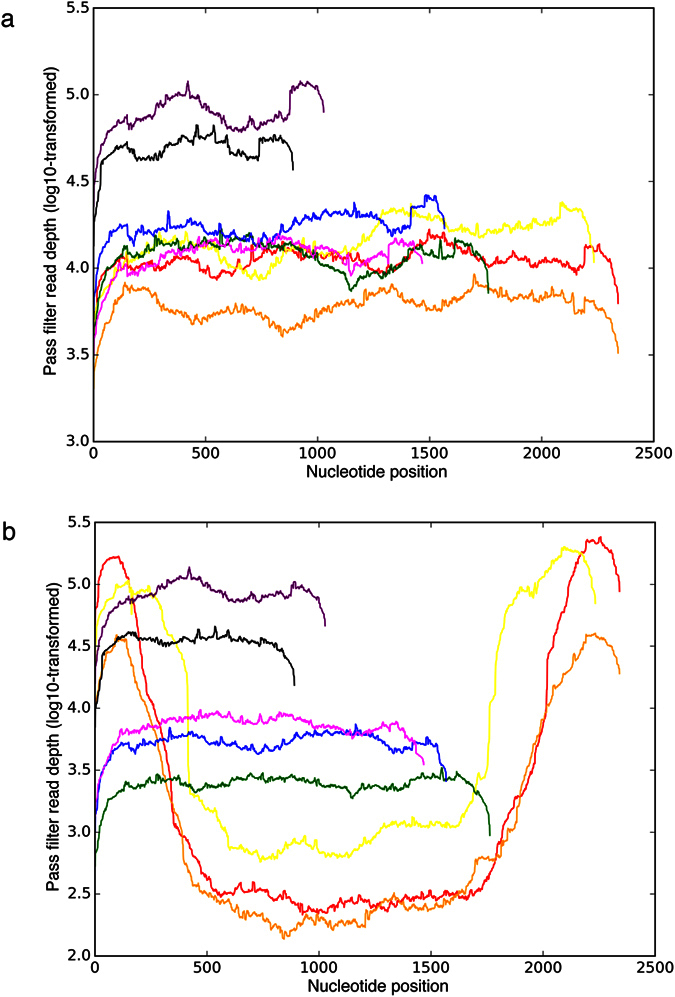
High-throughput sequencing results. (**a**) Ideal sequencing coverage from influenza A/Singapore/H2009.334C/2009(H3N2) virus genome. Red, orange, yellow, green, blue, magenta, purple, and black plotted lines represent sequencing coverage (X-axis) and pass filter read depth (Y-axis) of the influenza A/H3N2 segment 1 (PB2 - polymerase basic 2, 2341 nt), segment 2 (PB1 - polymerase basic 1, 2341 nt), segment 3 (PA - polymerase acidic, 2233 nt), segment 4 (HA – hemagglutinin, 1762 nt), segment 5 (NP – nucleoprotein, 1566 nt), segment 6 (NA – neuraminidase, 1467 nt), segment 7 (MP - matrix protein, 1027 nt), and segment 8 (NS – nonstructural, 890 nt), respectively. (**b**) Suboptimal sequencing coverage for influenza A/Singapore/C2009.803bV/2009(H3N2) virus genome. Considerably higher read depths were observed in the 5′- and 3′-ends of segments 1 (red), 2 (orange), and 3 (yellow).

This HTS protocol successfully obtained high read-depths covering the entire 5′- and 3′-ends of the 8 gene segments of influenza A/H3N2 viruses. However, the read depths per base were significantly different between gene segments. By including 16 samples in a run, the read depths per base reached as high as 119889 reads for the relatively short gene segment 7 (MP) and as low as 3183, 2027, and 2990 reads for the longer gene segments 1 (PB2), 2 (PB1), and 3 (PA), respectively (Fig. 3a). Depth of coverage for all the 148 influenza A/H3N2 viruses included in this study is shown in Supplementary Fig. S3.

### Sequence comparison between NGS and Sanger sequencing

In all, 34 base calls arising from 30 genes were identified as homogeneous (pure) base calls by HTS but were identified as either a different homogeneous (n = 18) or mixed (n = 16) base call by Sanger sequencing (Table 1). Re-examination of the Sanger chromatograms revealed that 15 discordant homogeneous base calls were incorrectly identified by the ATF contig assembler software version 1.0.2.41 (Connexio Genomics, Fremantle, Australia). The remaining 19 discordant base calls (representing 1.7% of all gene segment sequences) from HTS were found mainly near the 5′- and 3′-ends of the amplicons. That these discordant calls could also have been due to Sanger sequencing errors cannot be excluded.

**Figure 4:**
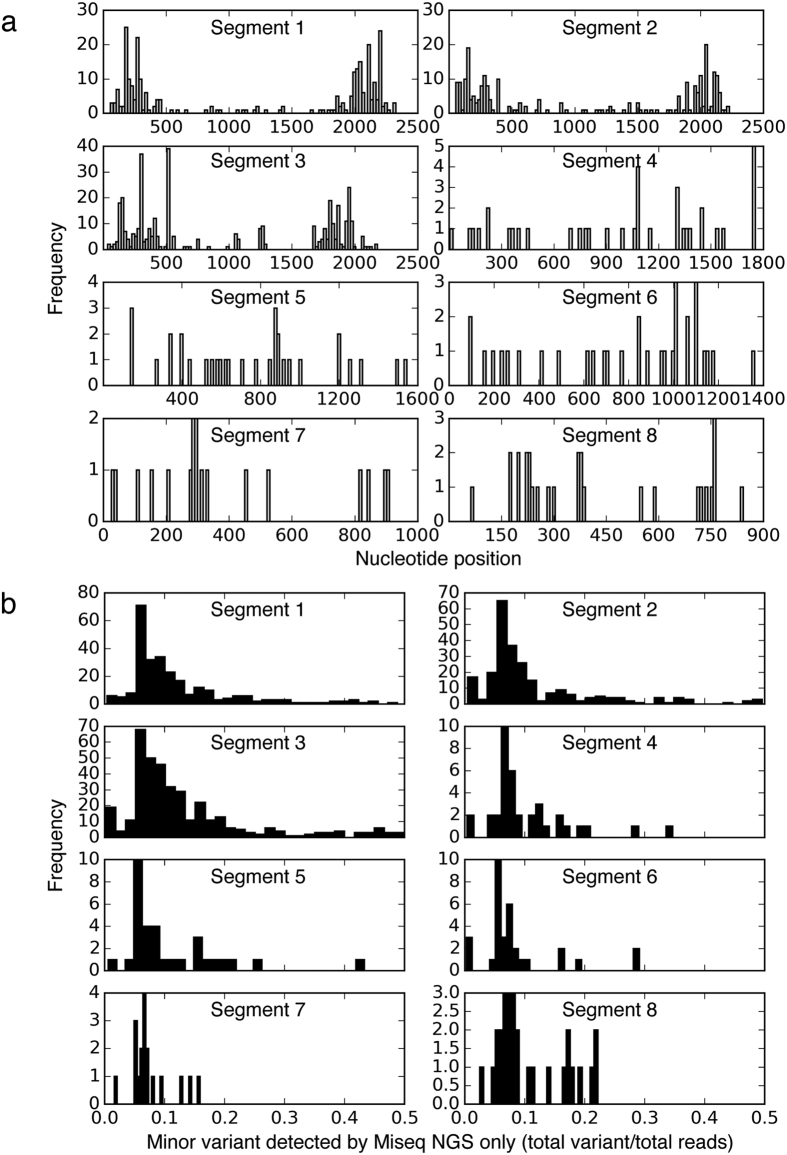
(**a**) Nucleotide positions of additional minor variants detected by high-throughput sequencing (HTS) only but not by Sanger sequencing in a total of 148 influenza A/H3N2 genome sequences. (**b**) Population frequencies of additional minor variants detected by HTS only.

In contrast, the HTS found an additional 1,025 mixed base calls that were absent in the results of the Sanger sequencing. Most of these 1,025 discordant mixed base calls belonged to the 3 longer gene sequences: segments 1/PB2 (270 detected from 75 sequences; 26%), 2/PB1 (247 detected from 65 sequences; 24%), and 3/PA (374 detected from 83 sequences; 36%). The majority of the additional minor variants detected in these 3 segments were found at the 5′- and 3′-ends of the segments (Fig. 4a).

These variants were mainly found in poor sequencing results that had extremely high reads clustered at the 5′- and 3′ends of the 3 segments (Fig. 3b). They accounted for 83% of the 270 PB2 minor variants (224 detected from 43 sequences), 82% of the 247 PB1 minor variants (202 detected from 39 sequences), and 76% of the 347 PA minor variants (283 detected from 38 sequences). The remaining 134 additional mixed calls were found in segments 4 to 8. Overall, most of the additional mixed variants detected by HTS in all gene segments represented minor populations of 5–20% frequencies (Fig. 4b).

### Variant quality

All the 15245 variants that passed the contamination filtering (i.e. upper limit of 99.99% prediction interval) had mapping quality of >40^20^,^21^, calculated by the GATK UnifiedGenotyper. Of the total variants, 13555 variants (89%) had strand bias FisherStrand (FS, an indicator of strand bias in sequencing) of <0.001. Of the remaining 1690 variants with FS >0.001, 99 (0.6%) had FS >60, which is the recommended threshold from GATK to determine if there is a significant strand bias between forward and reverse strands for the reference or alternate allele^20^,^21^. The higher the FS value, the more likely there is to be bias. All the 99 variants with FS >60 were obtained from the 5′- and 3′-end regions except 12 (12%) from the middle regions of PB2, PB1, and PA genes. Most of the variants located at 5′- and 3′-ends of the PB2, PB1, and PA genes had uneven clustered reads mentioned above (Fig. 3b). Nine of the 12 variants (75%) located at the middle region of the 3 genes had total read depth of <419, while the remaining 3 (25%) had read depths of 814, 1206, and 1246, respectively.

### Native sequencing error rate

To assess the native sequencing error rate, the sequences of the RNA replicase gene of the MS2 virus, amplified from the same sample in ten separate runs, were analyzed. Ten minor variants were consistently detected in the ten runs, with population frequencies ranging between 0.1% and 15.1%. The 10 variant nucleotide positions had total read depths ranging between 53032 and 442361 (median = 202124). They likely represent the true minority variants that are present in the sample. Another 4 minor variants were found only in 1–5 of the 10 runs, with total read depth of <40. Such read depths may be far too low to represent the native sequencing error. The 4 variants (i.e. nucleotide positions 2, 5, 1124, and 1125) were located <8 bp away from the 5′- and 3′-ends of the amplicons, and were part of the PCR primer sequences. The low read depths at the 5′- and 3′- ends of the amplicons were expected as they were sequenced using the original protocol, without the primer modifications for PCR amplification prior to Nextera XT library preparation.

## Discussion

### Even coverage amplicon sequencing

One of the key improvements of the amplicon-based HTS method described in this study was the successful sequencing of amplicons with even coverage across 5′- and 3′-ends of the amplicons, hitherto problematic regions of unacceptable low coverage prior to the modifications introduced. The direct integration of the transposon sequences to the Tuni-12 and Tuni-13 primer sequences during the initial genome-wide PCR negates the need for enzymatic addition of the transposon sequences by the transposase (Fig. 1b). This increases the read depths at both ends of the amplicons.

### Statistical exclusion of contamination reads

During the two-step PCR HTS library preparation of the Nextera XT DNA Library Prep kit, there is a high risk of cross-contamination during the set-up of the second PCR, especially by carry-over of the amplicons from the first PCR due to the high number of amplicons^10^. The amount of cross-contamination may vary due to different number of samples pooled during the library preparation step, and inter-operator variability. A semi-automated HTS library preparation protocol was optimized on the Sentosa SX101 liquid handling machine to control and minimize these errors.

To statistically exclude possible artefactual sequences caused by cross-contamination, an in-run NSC was included in each of the 10 library preparations to collect cross-contamination reads that may occur in different well positions. An upper limit of 99.99% prediction interval was determined based on the contaminating read depths observed in the NSCs of the 10 runs. Upon the initial amplicon dilution performed according to the library preparation protocol, tagmentation by the kit enzyme cleaves the diluted amplicons into fragment lengths of approximately 300 bp. Next, these fragments are subjected to the indexing PCR. All these steps may introduce between-sample contamination. The longest contaminating fragments or full-length amplicons (introduced during sample dilution) should fall within approximately 300 bp and above. Therefore, the artificial nucleotide replacements performed at every 150^th^ nucleotide starting from the first nucleotide of the reference sequence and subsequently called as a variant by the GATK variant caller should ensure that the contaminating noise was monitored across the whole segment, even though the base calls were initially perfectly matched with those of the reference sequence and may contain unacceptable read depths below the contamination cut-offs. Sequences with a base call (at any position surveyed) containing read depths below the background noise cut-off were excluded from further analysis. As such, the validity of variant calling for all nucleotide positions across the whole genome can be assured, rather than focusing only on certain mutational hotspots.

On a separate note, MS2 contaminating reads appeared to be more frequent in influenza samples that were placed right next to the NSC compared to ones that were placed at the far end from the NSC (data not shown). The entire HTS sequencing and bioinformatics protocol were tested on 8 RNA segments of influenza A/H3N2 viruses with different lengths (ranging between 890 and 2341 nucleotides) and genetic variability (ranging between 1.88 × 10^−3^ and 4.84 × 10^−3^ nucleotide substitutions/site/year)^22^,^23^. The substitution rate of 1 × 10^−3^ substitutions/site/year is found in many RNA viruses^24^. This study has successfully produced full-length sequences for segments 4 to 8 of influenza A/H3N2 viruses that were highly concordant with the sequences generated by Sanger sequencing method (Table 1).

### Clustered reads at termini sequences of the PB2, PB1, and PA genes

As with a previous study conducted on primary clinical samples of influenza A/H1N1/2009 pandemic virus^25^, a significant number of primary clinical samples of influenza A/H3N2 virus in this study may have a higher number of defective interfering particles (DIPs), producing clustered reads at 5′- and 3′-ends of the PB2, PB1, and PB2 genes (i.e. segments 1 to 3). Previous studies found that the influenza defective interfering RNAs typically retain the termini sequences of the PB2, PB1, and PA genes^25^,^26^. The relatively much higher HTS reads found for the MP gene (i.e. segment 7, Fig. 3a,b) in all H3N2 samples in this study may also be due to its importance in packaging full-length or truncated genome into viral-like particles, i.e. viable standard viral particles and/or DIPs^27^,^28^,^29^.

The clustered reads found at the 5′ and 3′ termini may also be due to sample degradation of the archived extracted RNA samples. All the RNA samples used in this study had been subjected to multiple freeze-and-thaw cycles previously, to carry out 19 PCRs for genome-wide amplification for Sanger sequencing^19^. The long PB2, PB1, and PA gene segments might have broken apart before further degradation. Re-extraction and re-sequencing from the archived raw clinical samples (nasal/nasopharyngeal/throat swabs in universal transport medium) for some of the problematic samples were able to resolve this problem (An example is shown in Supplementary Fig. 4). It is notable that the PA gene of such case did not have clustered reads at the 5′ and 3′ ends of the gene segment, suggesting a higher resistance to degradation in this gene. Lastly, the higher background noise detected at both the 5′- and 3′-ends may also be due to higher cross-contamination or higher biases/errors arising from PCR duplicates^30^ or errors generated during PCR extensions^31^,^32^.

### Strand bias

Similar to a previous study utilizing Nextera XT DNA Library Preparation kit, minimal strand bias was observed in this study^21^. The strand bias generally was limited to low read depth^21^ or 3′- and 5′-end regions with clustered high read depth of the PB2, PB1, and PA genes.

Briese *et al.*^33^ have recently showed potential genome-wide sequencing using virome capturing probes, without the initial genome-wide PCR prior to the HTS library preparation^33^. This could have efficiently reduced the sequencing artefacts found in this study. However, their work was conducted on a much higher sequencing throughput platform, the HiSeq 2500 (Illumina) instrument. Alternatively, mechanical shearing of the genome-wide amplicons followed by enzymatic ligation of sequencing adaptors should also result in even coverage throughout the whole genome. However, the latter approach would be more labor-intensive and would involve additional open-tube processing steps that may introduce a higher risk of between-samples contamination.

It is critical to understand the reliability of genome sequences generated by HTS methods, and under which circumstances the HTS-derived sequences may be acceptable without independent confirmation by Sanger sequencing^34^. Multiple studies have been performed to compare variant calling between HTS and Sanger sequencing methods^35^,^36^,^37^,^38^. However, few studies have compared whole-genome sequences generated by HTS to those produced by Sanger sequencing^39^. The pairwise comparison of genome sequences derived from current HTS and existing Sanger sequencing methods is complementary in phylogenetic analyses for viral sequences derived separately from both platforms. For instance, the current HTS data resolution for the HA or NA genes can be statistically reduced to match the Sanger sequencing data, i.e. to regenerate final consensus sequences by excluding minor variant lower than 20%, based on the statistics of difference found in Fig. 4b. It is well known that Sanger sequencing has a lower minor variant detection limit of 20%^40^,^41^.

Finally, it should be noted that this study quantified the overall contamination rate based on the sample processing/handling system of the current laboratory. The statistics of contamination is likely laboratory-dependent. Other laboratories performing HTS of influenza should establish their own thresholds for contaminant reads.

In conclusion, this study provides an estimate of the rate of specimen-to-specimen cross-contamination in clinical HTS testing. Clinical bioinformatics pipelines should incorporate robust contamination detection systems when interpreting HTS sequence data, particularly when dealing with low-frequency variants generated by amplicon-based HTS. This report also represents an attempt to address the requirements of the recent College of American Pathologists’ Laboratory Standard for Next-Generation Sequencing Clinical Tests, which stated the preference of giving laboratories performing HTS-based assays the flexibility to determine how a clinical test should be validated, but still addressing common problems that arise in the course of testing^34^.

## Methods

### Ethics statement

All research studies involving the use of these clinical samples were reviewed and approved by the local institutional ethics review board (National Healthcare Group: B/09/360 and E/09/341). All experiments were performed in accordance with the approved guidelines and regulations. This study only involved the use of archived, residual samples that were collected for routine diagnostic purposes. These samples were anonymized prior to analysis. The ethics committee exempted the requirement for written or verbal consent.

### Clinical samples

A total of 148 archived influenza A/H3N2 clinical samples collected from different patients between May 2009 and November 2013 were selected randomly for this study. All samples were received for diagnostic testing at the National University Hospital in Singapore and were confirmed positive using two clinically validated, in-house, real-time influenza A/B screening^17^ and subtyping assays^42^,^43^. The samples included nasal/nasopharyngeal/throat swabs collected in universal transport medium, endotracheal tube aspirates, or sputum samples.

### Preparation of MS2 bacteriophage dilution

A lyophilized MS2 bacteriophage (15597-B1) stock was commercially obtained from American Type Culture Collection, USA. The lyophilized stock was first re-suspended in 1 mL of carrier RNA (cRNA) solution, followed by a 10^4^-fold dilution using the cRNA solution. The cRNA solution was prepared from the Qiagen EZ1 Virus Mini Kit v2.0 (Qiagen, CA, USA), according to the manufacturer’s instructions. The cRNA reduces possible chance of viral RNA degradation by RNase molecules and enhances viral RNA recovery during sample extraction.

### Viral RNA extraction

Viral RNAs were extracted from 200 μL of clinical samples with the Qiagen EZ1 Virus mini kit v2.0 using the proprietary Bio Robot EZ1 automated platform (Qiagen), according to the manufacturer’s instructions. All extracted RNAs were eluted into a final volume of 60 μL of elution buffer. A similar extraction protocol was performed for the 10^4^-fold diluted MS2 viruses. To confirm the absence of MS2 RNA in the cRNA and/or the extraction kit, an extracted RNA that was not spiked with the MS2 virus was tested using a laboratory-developed real-time RT-PCR assay (Protocol will be provided upon request). The sample tested negative by the MS2 target-specific PCR.

### Reverse transcription polymerase chain reaction

The genome-wide RT-PCR amplification of the influenza A/H3N2 virus was performed using the Superscript III one-step RT-PCR system with Platinum *Taq* high-fidelity polymerase (Invitrogen, Carlsbad, CA). The 20-uL RT-PCR consisted of 10 μL of 2X Reaction Mix, 0.6 μmol/L of HFAdaptor, 0.6 μmol/L HRAdaptor, 0.4 μmol/L of HF, 0.4 μmol/L HR primers, 0.5 μL of enzyme mix, and 5 μL of RNA extract. The remaining volume was topped up with RNase-free water. The RT-PCR was initiated with an RT step of 42 °C for 30 min and a 2.5-min denaturation step of 95 °C, 5 amplification cycles consisting of 30 s at 95 °C, 45 s at 45 °C, and 3 min at 68 °C, 45 amplification cycles consisting of 30 s at 95 °C, 45 s at 60 °C, and 3 min at 68 °C, followed by a hold for 10 min at 68 °C as final extension.

Amplification of an 1130-nucleotide segment of the RNA replicase gene of the MS2 virus was prepared in 20-uL reaction volume, using the same Superscript III one-step RT-PCR kit mentioned above. The reaction volume consisted of 10 μL of 2X Reaction Mix, 0.3 μmol/L of MS2_1901F25 (5′-CTTAAGGGACGAATTGCTCACAAAG-3′; GenBank reference gene: NC_001417.2), 0.3 μmol/L of MS2_3006R25 (5′-GTGGATCTGACATACCTCCGACAAC-3′), 0.5 μL of enzyme mix, and 5 μL of RNA extract. The remaining volume was topped up with RNase-free water. The RT-PCR was initiated with an RT step of 55 °C for 15 min and a 2.5-min denaturation step of 95 °C, 5 amplification cycles consisting of 30 s at 95 °C, 30 s at 60 °C, and 75 s at 68 °C, followed by a hold for 10 min at 68 °C as final extension. All RT-PCRs were performed using either the ABI 9700 thermal cycler (Applied Biosystems, CA, USA) or the Biometra T3000 thermocycler (Biometra GmbH, Gottingen, Germany).

### DNA library preparation and high-throughput sequencing

The DNA library preparation was performed using the Nextera XT DNA Library Prep Kit (Illumina), according to the manufacturer’s protocol. The protocol was later adapted onto the Sentosa SX101 liquid handling machine for automation. Briefly, all PCR products were subjected to tagmentation, a step that enzymatically cleaved the DNA to fragments of approximately 300 bp in length and tagged the cleaved DNA with Illumina adapters. After the tagmentation, the cleaved DNA was subjected to a 12-cycle second PCR for indexing/barcoding with a unique combination of i5 and i7 index primers. After the indexing PCR, the PCR products were purified using Agencourt AMPure XP beads (Beckman Coulter Inc., CA, USA) and normalized using the Nextera XT Library Normalization Beads.

High-throughput sequencing was performed with MiSeq Reagent Kit v2 (300-cycle, Illumina) to generate 10–25 million 150-nucleotide paired-end reads per sequencing run, using the proprietary Illumina MiSeq System. Resequencing application under the Small Genome Sequencing category was selected to generate a sample sheet of the sequencing run, using the proprietary Illumina Experiment Manager. The default resequencing analysis settings were selected, i.e. flagging PCR duplicates and variant quality filter threshold of 30. A chloride wash was performed on the MiSeq sequencer before starting a new run, according to the manufacturer’s guide, to ensure minimal carry-over read contamination from the previous run.

### Bioinformatics

The bioinformatics analytic workflow was assembled using Python 3.4 and performed in CentOS 6.6 Linux operating system, using a workstation equipped with Dual Intel Xeon E5-2687W v2 3.4Ghz CPUs (16 cores) and 32-GB DDR3-1600 RAM. Briefly, the analytic workflow started with *de novo* assembly using VICUNA_v1.3 software^44^ on paired-end fastq files generated by MiSeq Reporter v2.6 (Illumina) or bcl2fastq2 Conversion Software v2.17 (Illumina). The sequence contigs were subjected to mapping against a local database consisted of all influenza A virus sequences available in the NCBI Influenza Virus Resource (Last accessed: 25 November 2015) to obtain the most matching reference gene segments, using blastn megablast task of BLAST 2.2.31+ software. Next, all degenerate sequences of the reference gene segments were replaced with non-degenerate sequences (i.e. “R” to “A”, “Y” to “C”, “S” to “G”, “W” to “A”, “K” to “G”, “M” to “A”, “B” to “C”, “D” to “A”, “H” to “A”, “V” to “A”, and “N” to “A”). Subsequently, each gene segment was annotated with a different nucleotide at every 150^th^ nucleotide position, starting from the first nucleotide of the sequence (i.e. “A” to “T” and “T/G/C” to “A”).

Paired-end alignments were performed using the aln and sampe modules of the BWA-0.6.2 software^45^, against the influenza A/H3N2 reference genome, followed by variant annotation using the UnifiedGenotyper module of GATK-3.4–46^14^,^15^,^16^. Reads or fragment of reads with quality score below 15 were trimmed during bwa alignment. A minimal phred-scaled confidence threshold of 10 at which variants should be called by the GATK was used. Instead of using the quality score calculated by the GATK, the upper limit of 99.99% prediction interval of contamination reads was used as the cut-off for calling an absolute or mixture base. A detailed description of the workflow and program scripts can be downloaded from http://hkailee.github.io/FluSeq.

### Exclusion of erroneous variant calling that may be due to contamination

In-house developed python scripts were used to filter the variant calls by considering an upper limit of 99.99% prediction interval of background/contaminating reads. The upper limit for 99.99% prediction intervals for the average value of Y_0_ (expected contamination/background reads) for a given X_0_ (total amount of pass filter reads for a sample), is


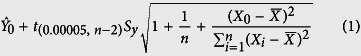


where S_y_ is the standard deviation of the residuals, calculated as


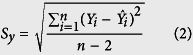


Final consensus sequences were generated based on filtered variant annotations and the reference genome.

### Pairwise comparison with Sanger sequencing results

The generated consensus sequences were compared against Sanger-generated sequences. We have previously published the genome sequences of the 148 H3N2 primary clinical samples derived from our Sanger sequencing method^19^. Most of the influenza genome was covered bi-directionally by Sanger sequencing reads with high quality values to ensure accurate base-calling^19^. Forward and/or reverse sequencing were repeated for ambiguous mixed base results (i.e. the second highest chromatography signal peak height (minor variant) lower than 25% of the highest peak height (major variant)), which were either present in only forward or reverse sequencing read or the ambiguous region was only covered uni-directionally.

## Additional Information

**How to cite this article**: Lee, H. K. *et al.* Contamination-controlled high-throughput whole genome sequencing for influenza A viruses using the MiSeq sequencer. *Sci. Rep.* **6**, 33318; doi: 10.1038/srep33318 (2016).

## Supplementary Material

Supplementary Information

Supplementary Dataset 1

Supplementary Dataset 2

## Figures and Tables

**Table 1:** Summary of the total variants for each gene segment before and after background filtering and pairwise comparison between high-throughput sequencing- (HTS-) and Sanger-derived full-length sequences.

Segment/Gene /Length	Background filtering								
	Before	After	Pairwise sequence comparison between HTS and Sanger						
	Total variants (minor + absolute) /No. of sequences	Total variants (minor + absolute) /No. of sequences	Total sequences compared	Total minor variants found by HTS after filtering /No. of HTS sequences	Total minor variants found by Sanger /No. of Sanger sequences	No. of fully concordant sequences	Total additional minor variants found by HTS /No. of sequences	Total homogeneous variants compared to Sanger method after filtering /No. of sequences	Total homogeneous variants correctly identified by HTS after re-visiting Sanger chromatogram/No. of sequences
1/PB2/2341nt	2968/148	2511/130	129	403/129	133/129	53 (41%)	270/75	0/0	0
2/PB1/2341nt	3035/148	2532/130	130	377/128	132/130	60 (46%)	247/65	9/8	2/2
3/PA/2233nt	2956/148	2641/136	135	513/136	138/135	49 (36%)	374/83	6/5	4/3
4/HA/1762nt	2016/148	1860/138	138	175/136	140/137	105 (76%)	37//30	3/3	1/1
5/NP/1566nt	1949/148	1839142	142	172/138	147/142	112 (79%)	32/26	7/5	4/2
6/NA/1467nt	1748/148	1638/140	140	173/138	144/140	115 (82%)	32/21	4/4	2/2
7/MP/1027nt	1206/148	1155/142	142	163/142	146/142	126 (89%)	18/15	1/1	1/1
8/NS/890nt	1114/148	1069/143	143	175/140	150/143	117 (82%)	28/22	4/4	1/1
