# Supplementary material for: Contamination-controlled high-throughput whole genome sequencing for influenza A viruses using the MiSeq sequencer
Source: Sci Rep. 2016 Sep 14;6:33318. doi: 10.1038/srep33318 (PMC5022032; doi:10.1038/srep33318)
Supplement: Supplementary Information [file srep33318-s1.doc]

**Contamination-controlled high-throughput whole genome sequencing for influenza A viruses using the MiSeq sequencer**

Hong Kai Lee^1*^, Chun Kiat Lee^1^, Julian Wei-Tze Tang^2,3^, Tze Ping Loh^1^, and Evelyn Siew-Chuan Koay^1,4^

^1^Department of Laboratory Medicine, National University Hospital, National University Health System, Singapore; ^2^Department of Infection, Immunity, Inflammation, University of Leicester, Leicester, UK; ^3^Clinical Microbiology, Leicester Royal Infirmary, Leicester, UK; ^4^Department of Pathology, Yong Loo Lin School of Medicine, National University of Singapore, Singapore


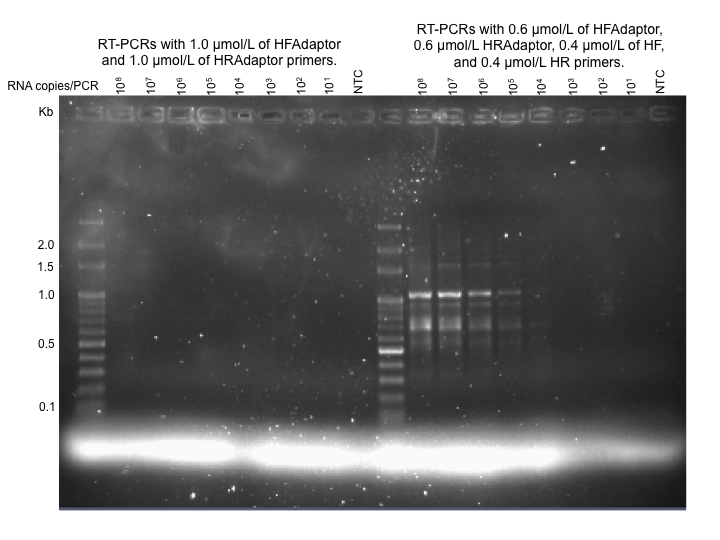
**Supplementary Figure S1.** Genome-wide reverse transcription polymerase chain reactions using RNAs diluted serially in 10-fold (10^1^ - 10^8^ copies/PCR) from a high viral titre clinical sample. The segment 1 (PB2 - polymerase basic 2), segment 2 (PB1 - polymerase basic 1), segment 3 (PA - polymerase acidic), segment 4 (HA – hemagglutinin), segment 5 (NP – nucleoprotein), segment 6 (NA – neuraminidase), segment 7 (MP - matrix protein), and segment 8 (NS – nonstructural), have sizes of 2341, 2341, 2233, 1762, 1566, 1467, 1027, and 890 nt, respectively. A faint band of MP gene (1027 nt) can be merely visualized in 10^8^ copies/PCR of the protocol with only HFAdaptor and HRAdaptor primers (Left). In contrast, the lower sensitivity limit of the optimized protocol (Right) falls at 10^4^ copies/PCR.


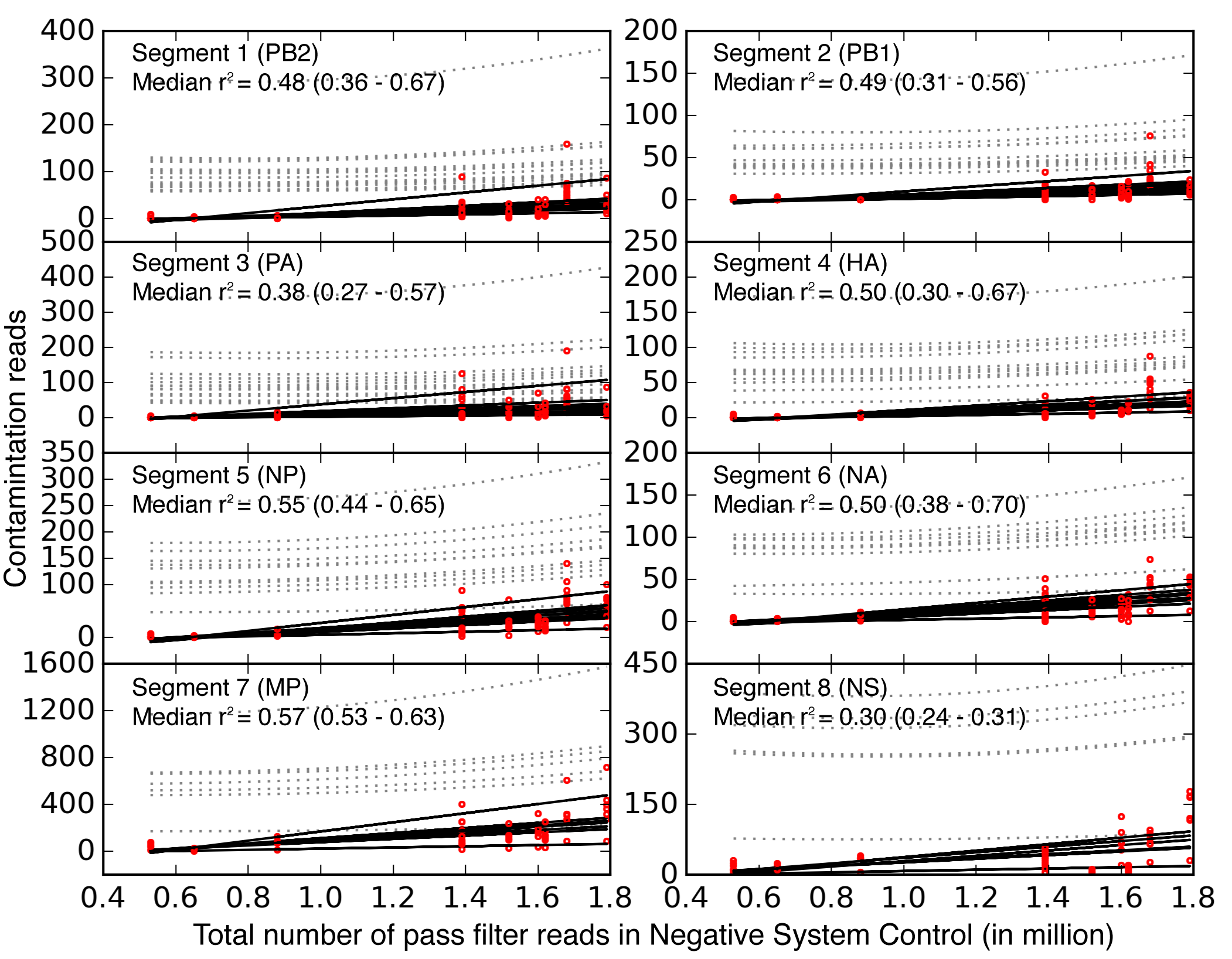


**Supplementary Figure S2.** Scatter plots (red dots) for number of background/contamination reads found in the Negative System Controls (NSCs) included in the 10 separate runs, recorded every 150^th^ nucleotide position starting from first nucleotide onwards along the 8 different gene segments, according to the numbers of total pass-filter reads belonging to the NSCs. The solid and dashed lines represent trendlines and 99.99% upper prediction limits of number of contamination reads found in each nucleotide position surveyed.

a)


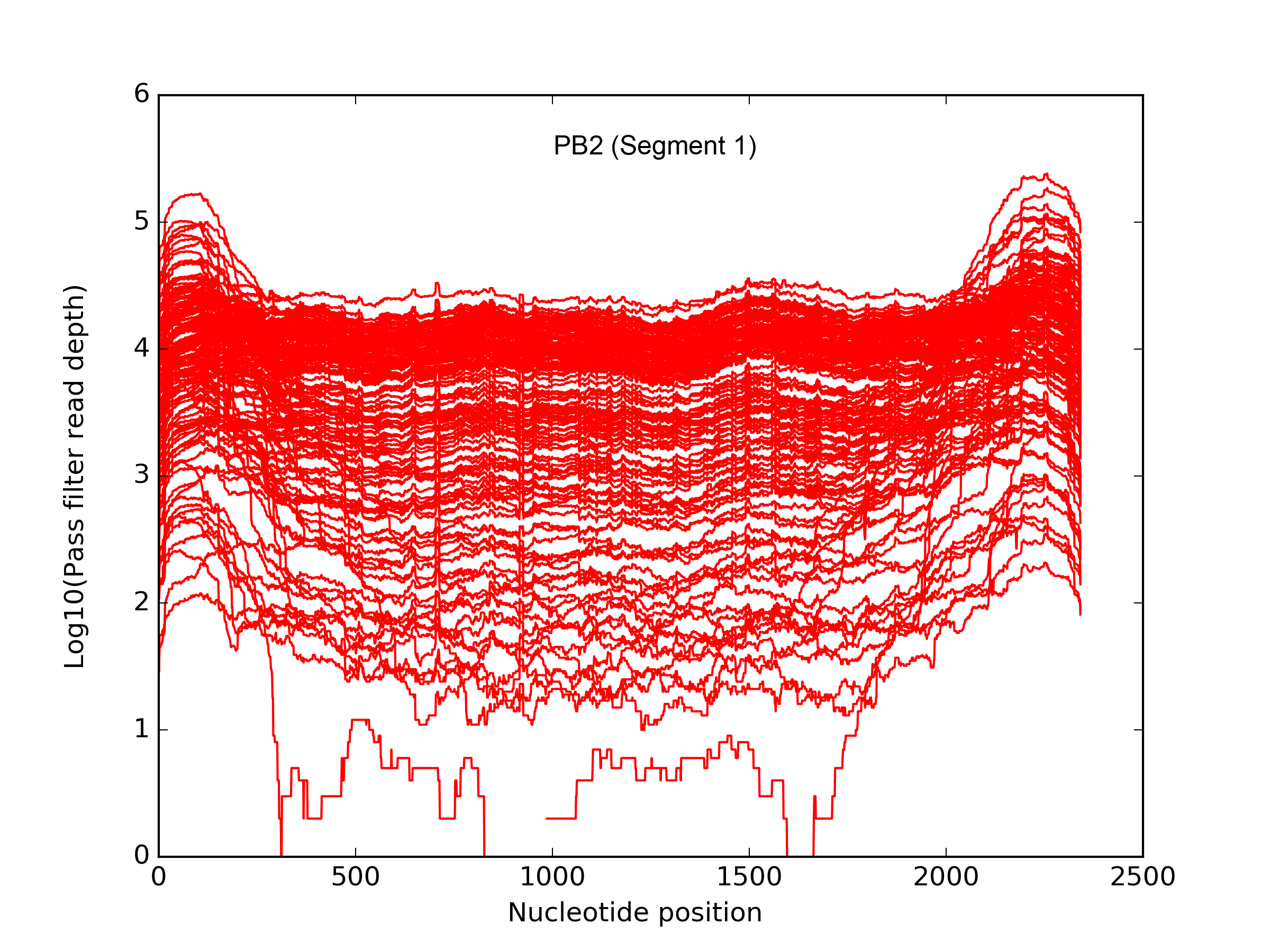


b)


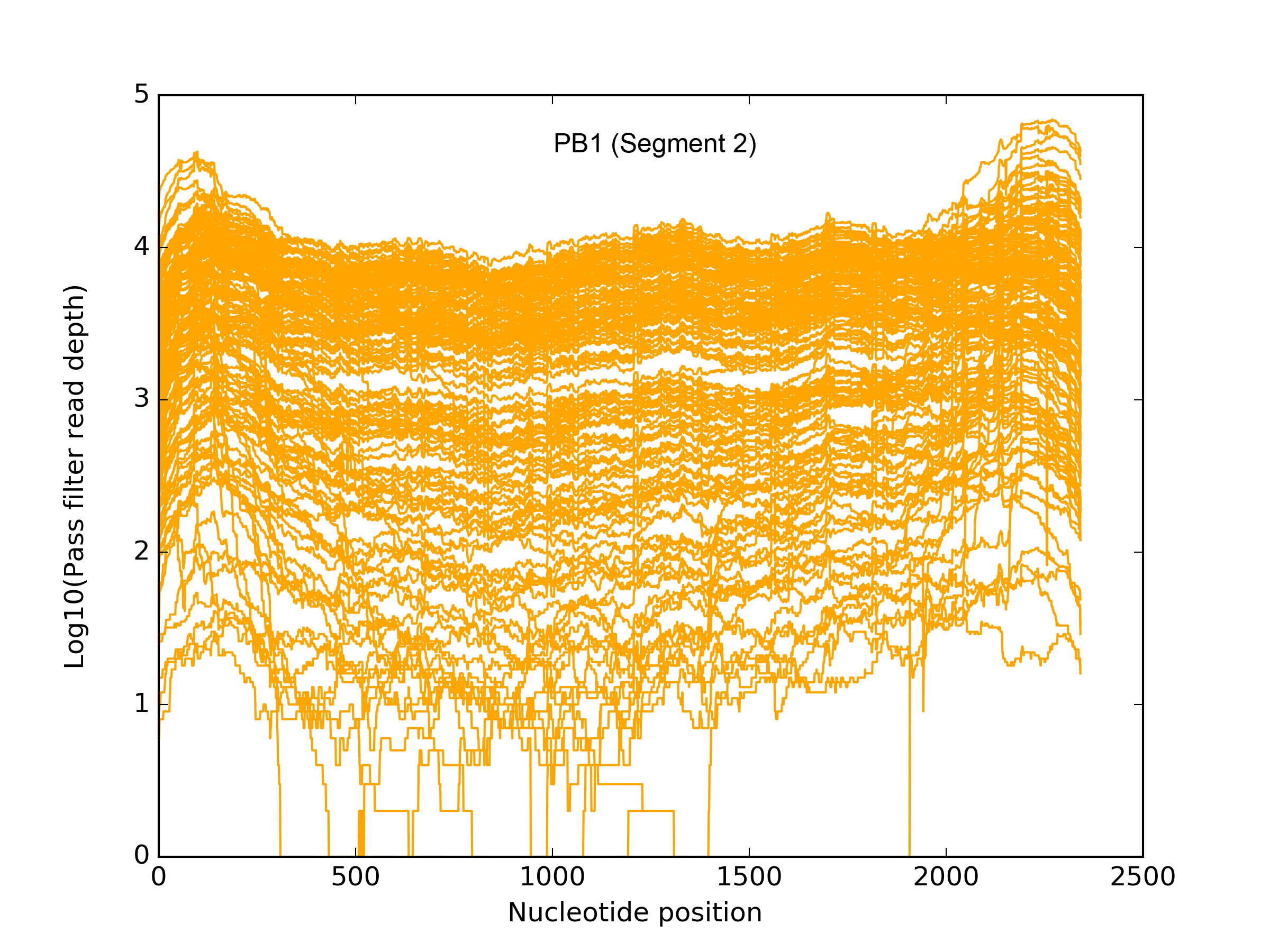


c)


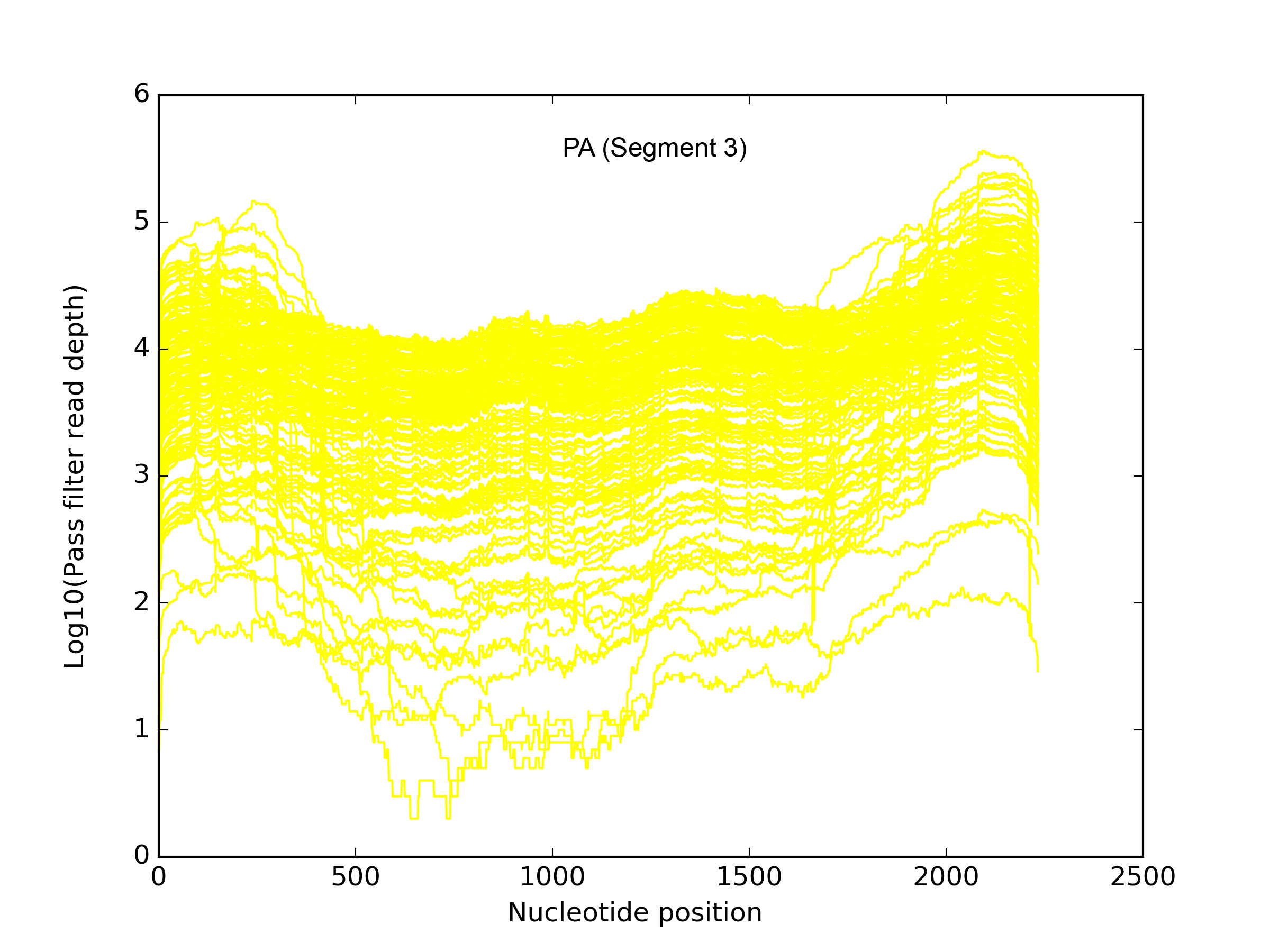


d)


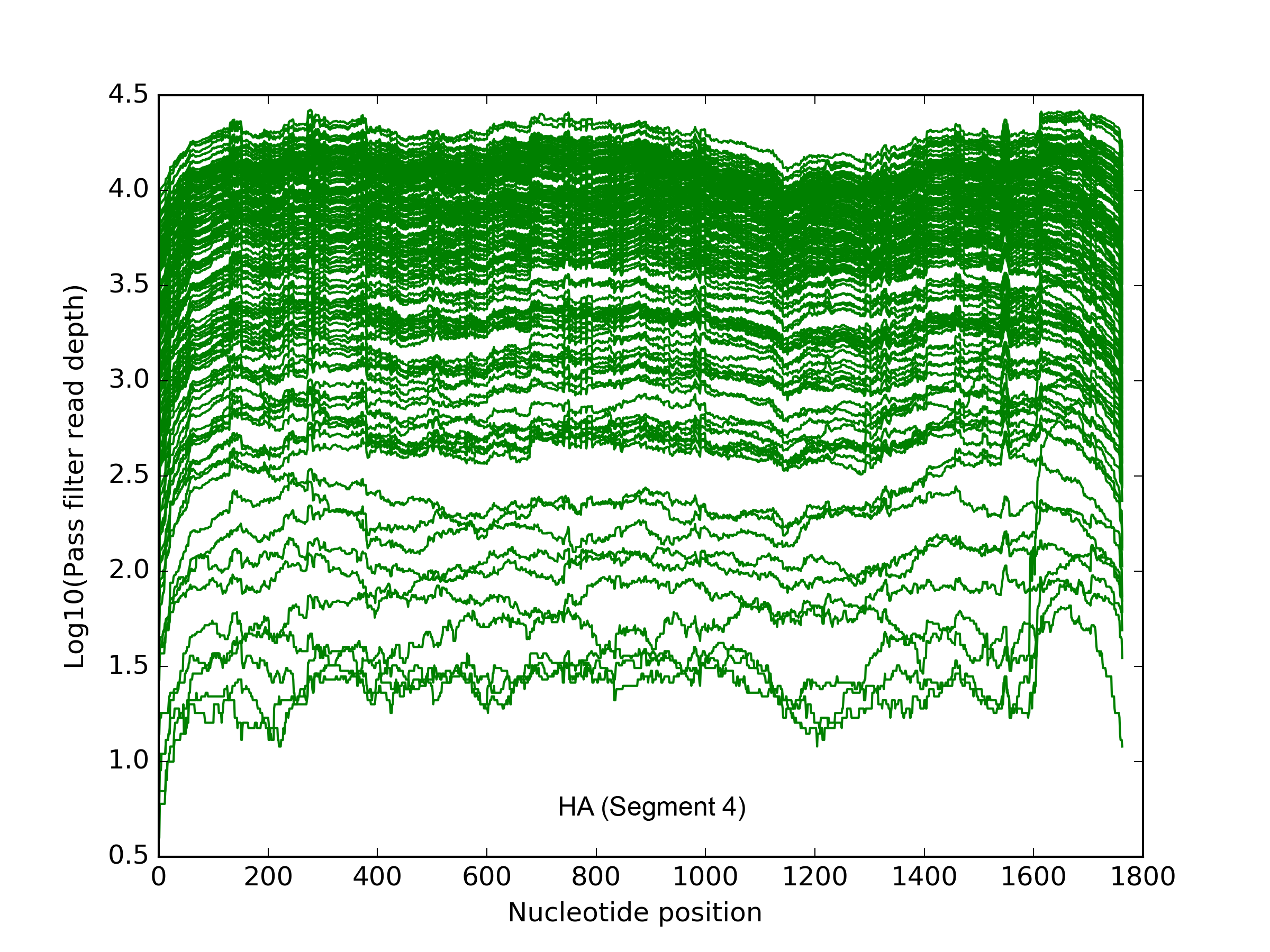


e)


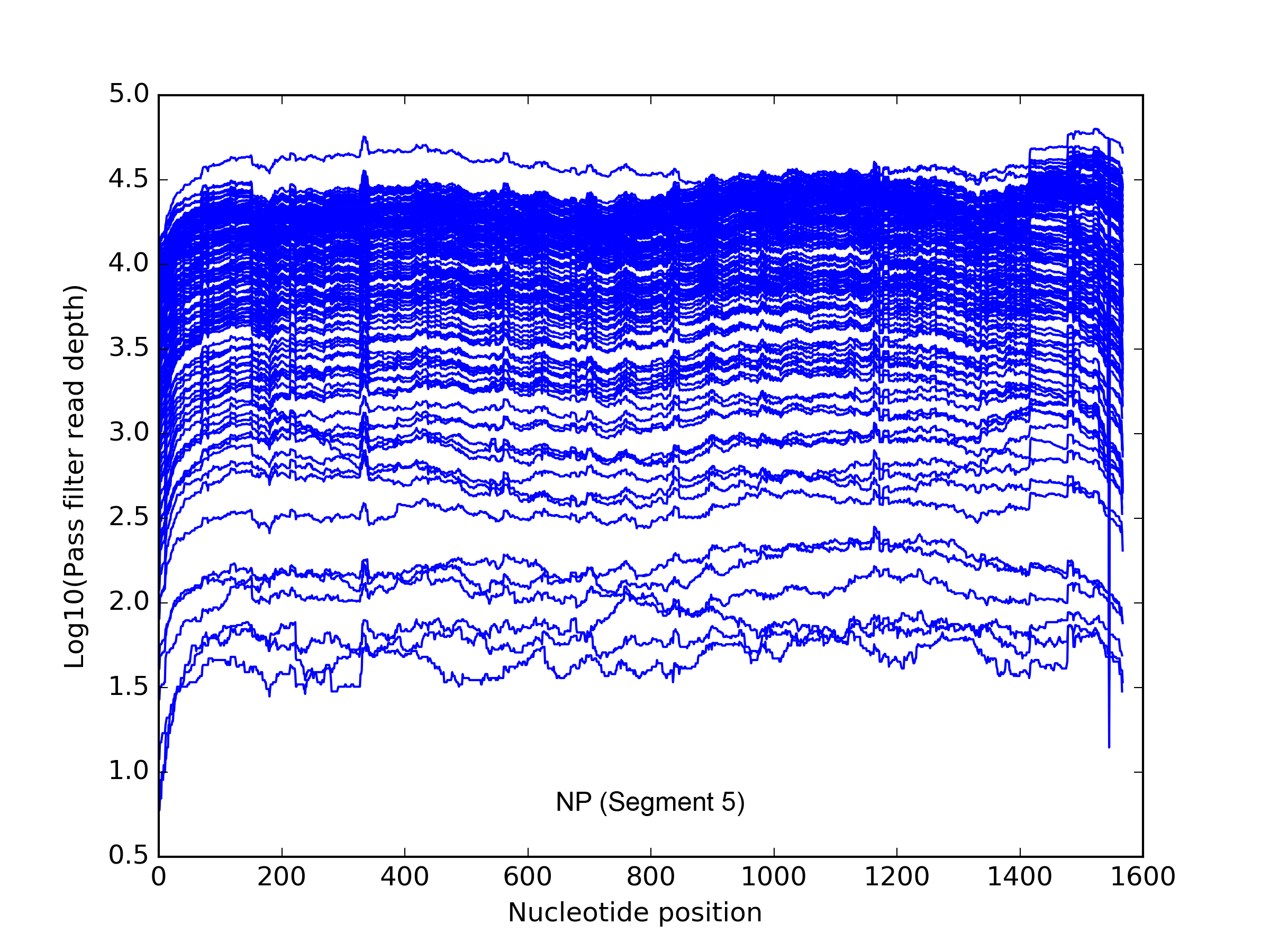


f)


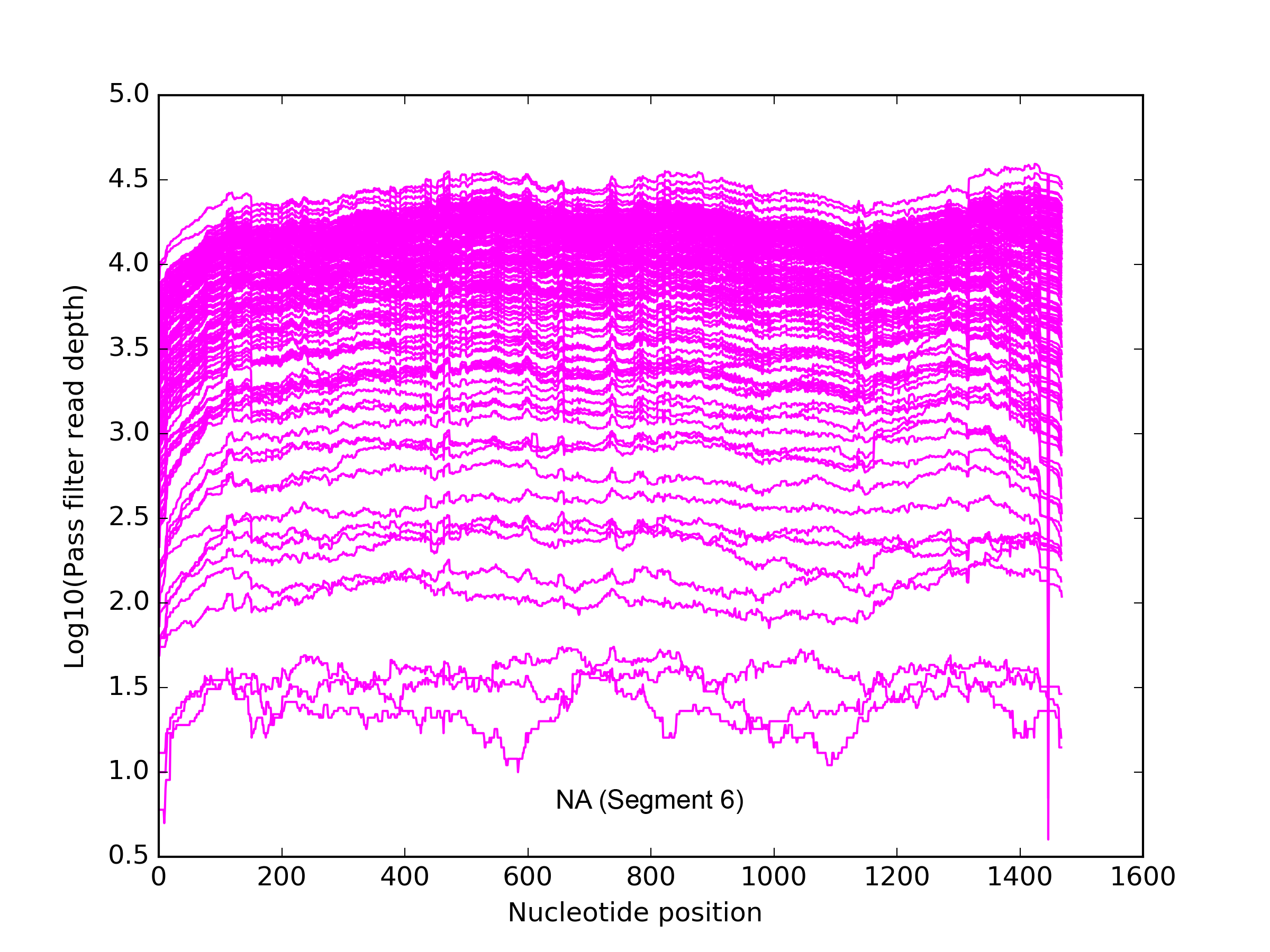


g)


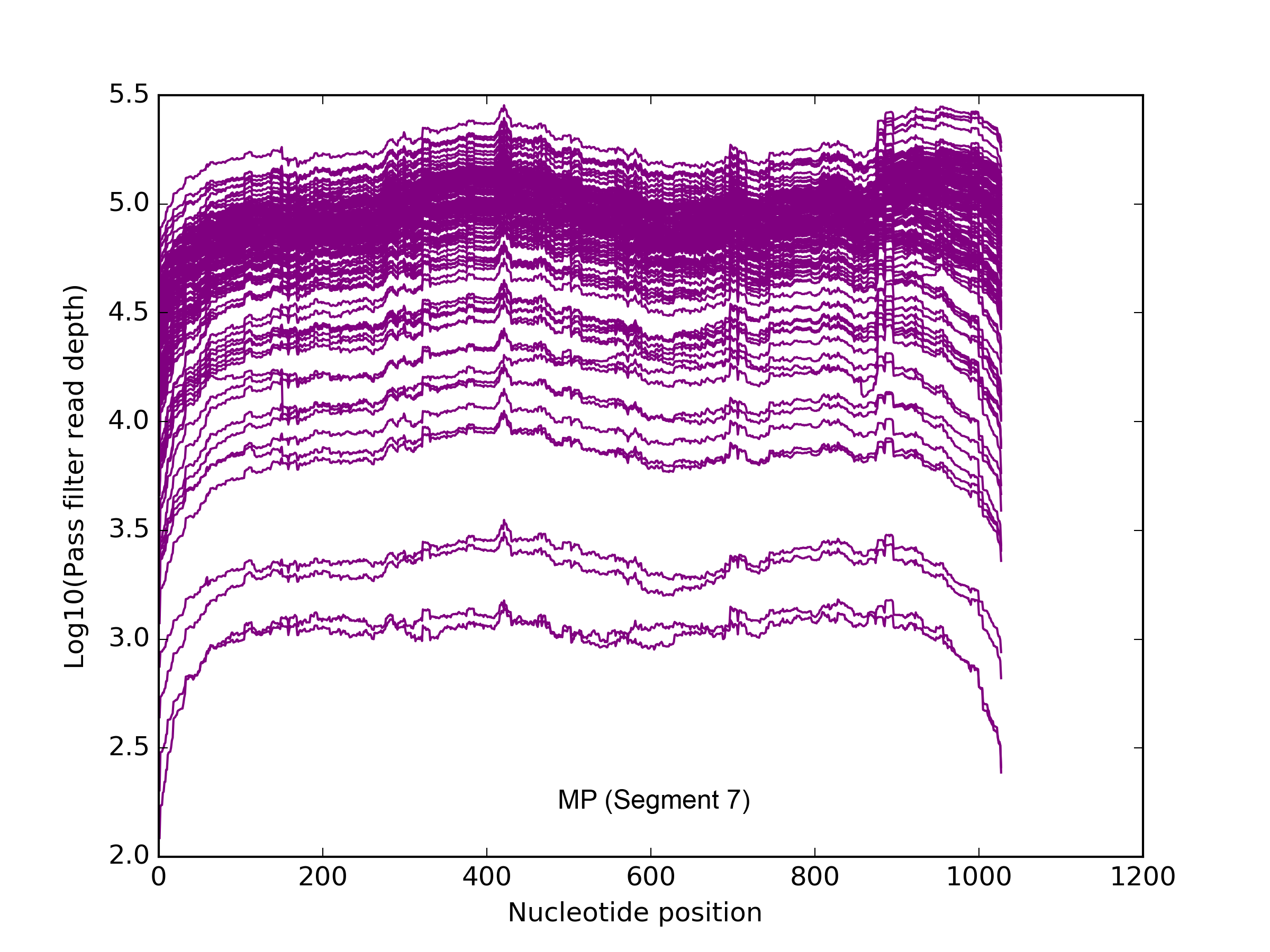


h)


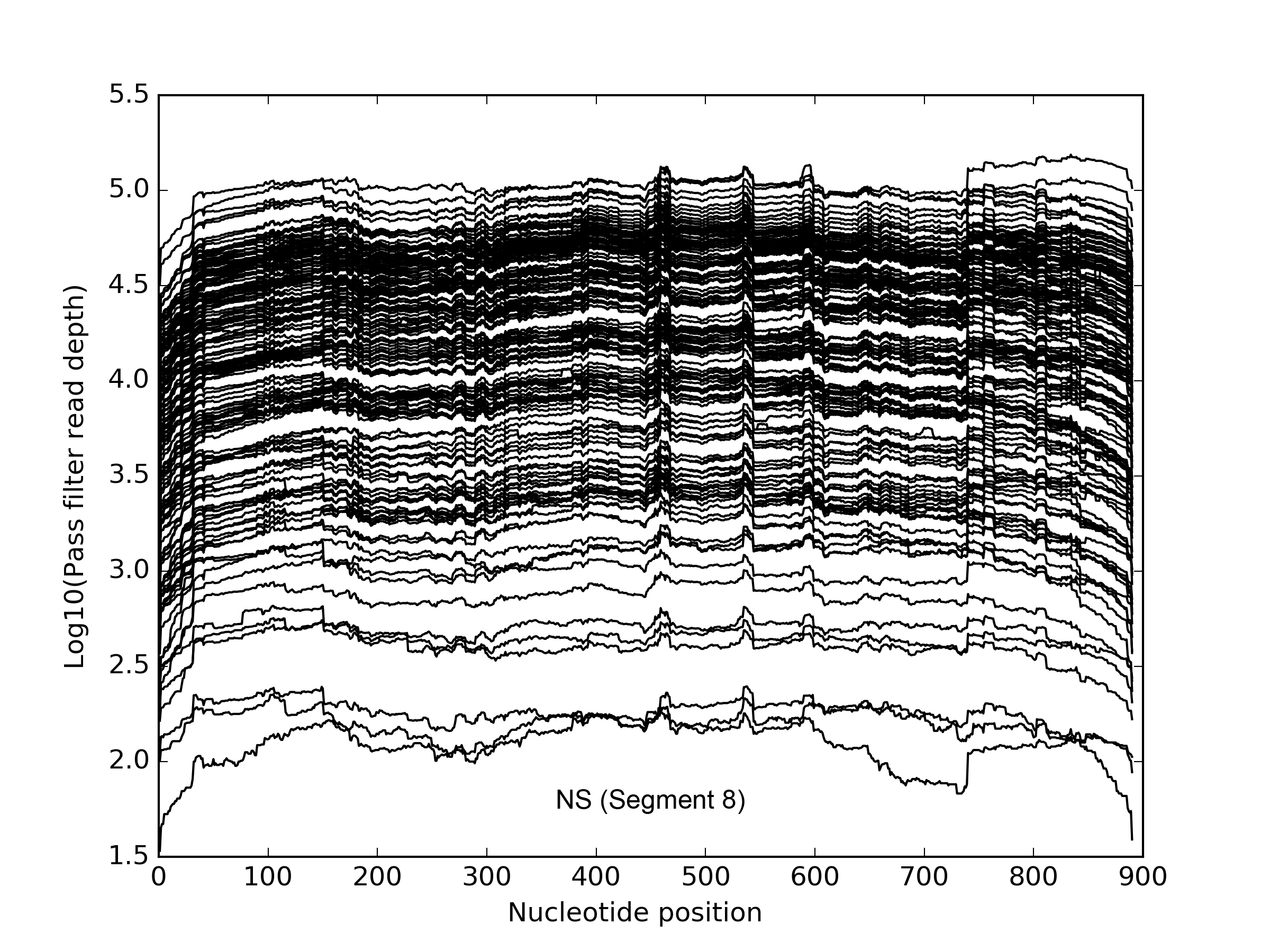


**Supplementary Figure 3.** Depth of coverage for high-throughput sequencing of all the 148 influenza A/H3N2 viruses included in this study. Red, orange, yellow, green, blue, magenta, purple, and black plotted lines represent sequencing coverage (X-axis) and pass filter read depth (Y-axis) of the influenza A/H3N2 a) segment 1 (PB2 - polymerase basic 2, 2341 nt), b) segment 2 (PB1 - polymerase basic 1, 2341 nt), c) segment 3 (PA - polymerase acidic, 2233 nt), d) segment 4 (HA - hemagglutinin, 1762 nt), e) segment 5 (NP – nucleoprotein, 1566 nt), f) segment 6 (NA – neuraminidase, 1467 nt), g) segment 7 (MP - matrix protein, 1027 nt), and h) segment 8 (NS – nonstructural, 890 nt), respectively. All the gene segments achieved high read depths across the 5’- and 3’-ends of the sequences. Considerably higher read depths were observed in 5’- and 3’-ends of segments 1, 2, and 3.


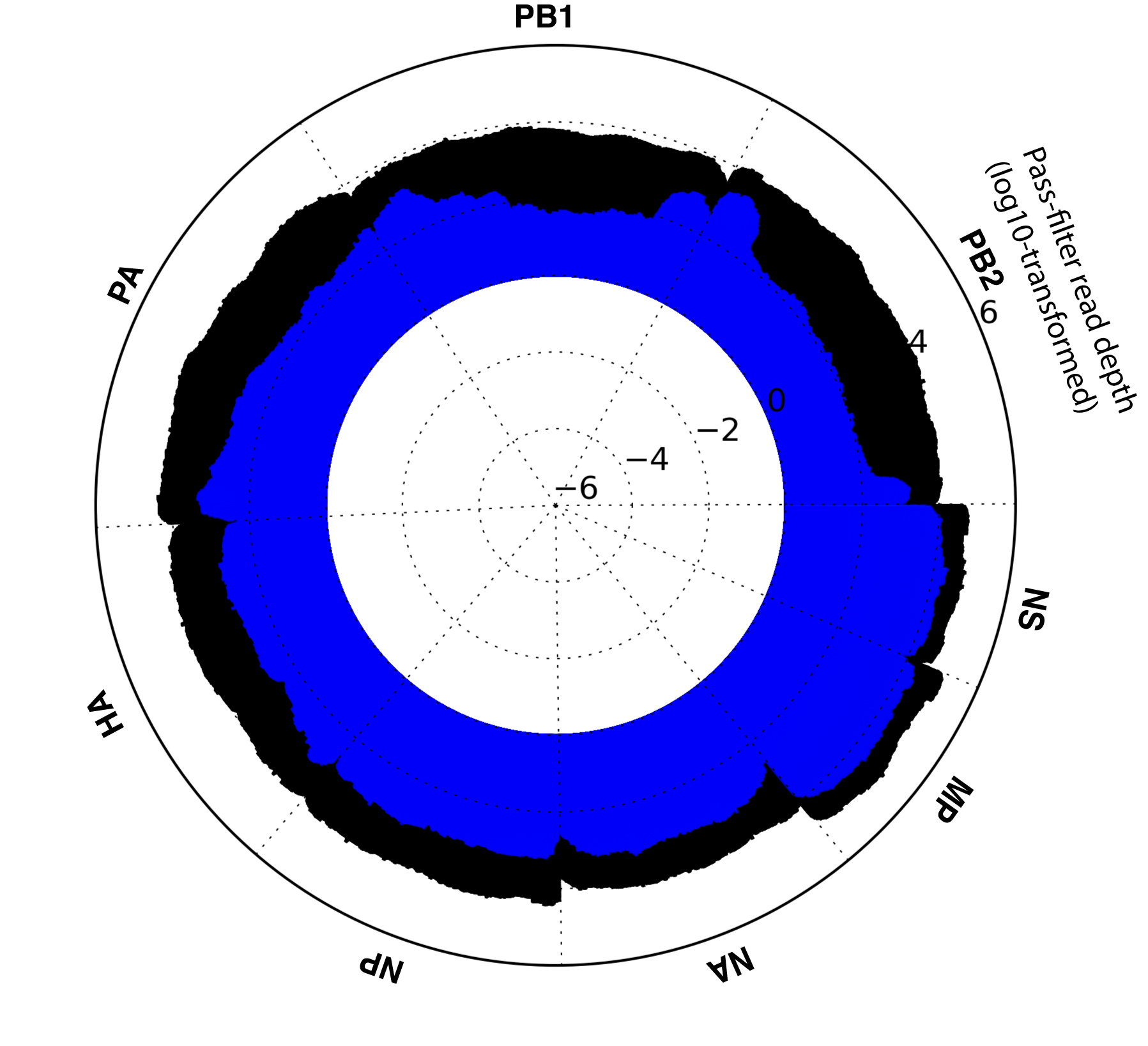


**Supplementary Figure 4.** Depth of coverage for high-throughput sequencing for influenza A/Singapore/H2009.334C/2009 strain included in this study. The blue bars show the depth of coverage for genome of an archived RNA sample with clustered reads found at 5’ and/or 3’ ends of polymerase basic 2 (PB2), PB1, and polymerase acidic (PA) genes. The black bars show an even depth of coverage for genome of the corresponding re-extracted RNA sample, including the PB2, PB1, and PA genes.
